# Reactive Hyperplastic Lesions of the Oral Cavity

**Published:** 2015-03

**Authors:** Hamideh Kadeh, Shirin Saravani, Mohammad Tajik

**Affiliations:** 1 *Dental Research Center, School of Dentistry, Zahedan University of Medical Sciences, Zahedan, Iran.*; 2 **School of Dentistry, Zahedan University of Medical Sciences, Zahedan, Iran.**

**Keywords:** Fibroma, Oral cavity, Pyogenic granuloma, Soft tissue

## Abstract

**Introduction:**

Peripheral reactive lesions of soft tissue are common oral lesions that dentists face during routine examinations. Diagnosis and development of a treatment plan is difficult if dentists are not aware of the prevalence and clinical symptoms of these lesions. The frequency of these lesions differs across various populations. The aim of this study was to determine the frequency and distribution of oral reactive lesions over a period of 7 years (2006–2012).

**Materials and Methods:**

In this retrospective study, available records from the archives of the Department of Pathology, Dental School and the two main hospitals in southeast of Iran (Zahedan) over a period of 7 years (2006–2012) were reviewed. Information relating to the type of reactive lesion, age, gender and location was extracted and recorded on data forms. Data were analyzed using SPSS statistical software (V.18) using the chi-square and Fisher’s exact test.

**Results:**

Of 451 oral lesions, 91 cases (20.2%) were reactive hyperplastic lesions. The most common lesions were pyogenic granuloma and irritation fibroma, respectively. These lesions were more frequent in women (60%) than men (40%). The most common locations of involvement were the gingiva and alveolar mucosa of the mandible, and lesions were more common in the 21–40-year age group. The relationship between age group and reactive lesions was statistically significant (P=0.01).

**Conclusion:**

The major findings in this study are broadly similar to the results of previous studies, with differences observed in some cases. However, knowledge of the frequency and distribution of these lesions is beneficial when establishing a diagnosis and treatment plan in clinical practice.

## Introduction

Because the oral mucosa is constantly under the influence of various internal and external stimuli, it exhibits a range of developmental disorders, irritation, inflammation, and neoplastic conditions (1). Reactive lesions are tumor-like hyperplasias which show a response to a low-grade irritation or injury (2), such as chewing, food impaction, calculus, iatrogenic injuries such as broken teeth, overhanging dental restorations and extended flanges of denture (3). Irritation fibroma, pyogenic granuloma, peripheral giant cell granuloma, and cemento-ossifying fibroma are common oral cavity reactive lesions. Other reactive lesions of the oral cavity include epulis fissuratum, inflammatory papillary hyperplasia and inflammatory fibrous hyperplasia (2). Reactive lesions are commonly seen in the gingiva and their occurrence in other places of the oral cavity, such as the tongue, palate, cheek and floor of the mouth is less common (1). Clinical features of these lesions consist of sessile or pedunculated masses with smooth or injured surfaces, and are seen in different colors, from bright pink to red (4). Since it is possible to detect lesions with a specific nature based on their histopathological features, these lesions can be divided into vascular and fibrous types (5). Various studies have reported differences in the type of reactive lesions, age distribution, gender, location, and clinical behavior of these lesions in different populations (2). The clinical appearance of these lesions is similar to neoplastic lesions. This similarity is a challenge in the process of diagnosis (2). Furthermore, early detection and treatment of reactive lesions by dentists can reduce dentoalveolar complications. Therefore awareness of the frequency and description of such lesions can help clinicians to make a better diagnosis and offer optimal treatment (6). Studies of the frequency of oral cavity reactive lesions have not previously been conducted in Southeast Iran. The aim of this retrospective study was to determine the relative prevalence and distribution of the oral cavity reactive lesions referred to the Department of Oral Pathology and two hospitals in Zahedan (Southeast of Iran) during a 7-year period and to compare the results with those of similar studies.

## Materials and Methods

In this retrospective study, records in the Department of Oral Pathology, Dental School, Zahedan University of Medical Sciences and Khatam-Alanbia and TaminEjtemaei Hospitals were extracted between 2006 and 2012. Records with a histopathological diagnosis of reactive hyperplasic lesions of the oral cavity were selected. These lesions were classified into two groups: fibrous lesions with connective tissue predominantly consisting of collagen (epulis fissuratum, irritation fibroma, giant cell fibroma, peripheral ossifying fibroma) and soft hemorrhagic lesions that are highly vascular, in which hemorrhage is an important clinical and histological feature (pyogenic granuloma, peripheral giant cell granuloma, epulis granulomatosum and pregnancy tumor) (5).

The only exclusion criterion was incompletely registered records. Clinical data regarding age, gender, and the anatomical location of the lesions were collected for each case. Data were analyzed using SPSS software version 18.0 (SPSS Inc, Chicago, IL) using descriptive statistical methods (means, standard deviations and percentages), chi-square and Fisher’s exact test. P-values less than 0.05 were considered statistically significant.

## Results

**Fig 1:**
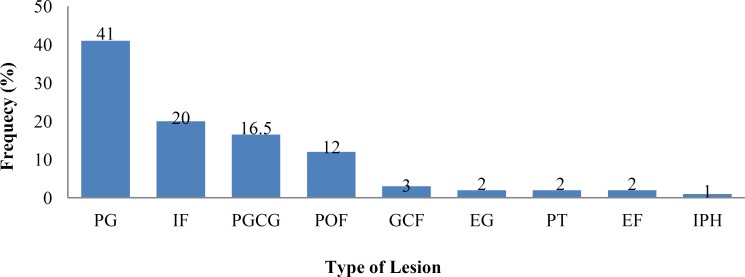
Distribution of oral cavity reactive lesions

Of a total of 451 lesions recorded in the biopsy records during the period assessed, 91(20.2%) were reactive hyperplasia; 35(38%) were fibrous lesions and 56 (62%) were soft hemorrhagic lesions. The most common lesion was pyogenic granuloma (n=37, 41%) followed by 18 cases (20%) of irritation fibroma, 15 cases (16.5%) of peripheral giant cell granuloma, 11 cases (12%) of peripheral ossifying fibroma, and the least common lesion was inflammatory papillary hyperplasia (n=1, 1%). The most prevalent reactive hemorrhagic and fibrous lesions were pyogenic granuloma and irritation fibroma, respectively (Fig. 1).

PG: Pyogenic granuloma, IF: Irritiation fibroma, PGCG: Peripheral giant cell granuloma, POF: Peripheral ossifying fibroma, GCF: Giant cell fibroma, EG: Epulis granulomatosum, PT: Pregnancy tumor, EF: Epulis fissuratum, IPH: Inflammatory papillary hyperplasia

The mean age of the patients in this study was 33.95±17.75 years (range, 4–88 years). The mean age of patients with irritation fibroma (IF), pyogenic granuloma (PG), peripheral ossifying fibroma (POF) and peripheral giant cell granuloma (PGCG) were 42.5±23.4, 30.4±14.9, 41.3±15.1 and 25.6±16.2 years, respectively. The lesions were most commonly seen in the third and fourth decades of life and less commonly seen above the age of 60 years. 

Fibrous lesions were more common in the fifth and sixth decades and soft hemorrhagic lesions were more common in the third and fourth decades of life. 

The distribution of fibrous and hemorrhagic lesions in relation to age group is shown in Tables 1 and 2. The relationship between age group and fibrous/ hemorrhagic lesions was statistically significant (P=0.012).

**Table 1:** Distribution of fibrous lesions by age group

**Lesion**	**IF**	**POF**	**GCF**	**EF**	**IPH**	**Total**
Age group	No. (%)	No. (%)	No. (%)	No. (%)	No. (%)	No. (%)
≤20	4 (4.5)	2 (2)	0	1 (1)	0	7 (7.5)
21–40	4 (4.5)	3 (3.5)	1 (1)	0	0	8 (9)
41–60	6 (6.5)	4 (4.5)	2 (2)	1 (1)	1 (1)	14 (15)
≥61	4 (4.5)	2 (2)	0	0	0	6 (6.5)
Total	18 (20)	11 (12)	3 (3)	2 (2)	1 (1)	35 (38)

IF: Irritation fibroma, POF: Peripheral ossifying fibroma, GCF: Giant cell fibroma, EF: Epulis fissuratum, IPH: Inflammatory papillary hyperplasia

**Table 2:** Distribution of soft hemorrhagic lesions by age group

**Lesion**	**PG**	**PGCG**	**PT**	**EG**	**Total**
Age group	No. (%)	No .(%)	No. (%)	No. (%)	No. (%)
≤20	11 (12)	7 (7.5)	0	0	18 (19.5)
21–40	15 (16.5)	5 (5.5)	2 (2)	2 (2)	24 (26)
41–60	10 (11.5)	3 (3)	0	0	13 (14.5)
≥61	1 (1)	0	0	0	1 (1)
Total	37 (41)	15 (16.5)	2 (2)	2 (2)	56 (61)

PG: Pyogenic granuloma, PGCG: Peripheral giant cell granuloma, PT: Pregnancy tumor, EG: Epulis granulomatosum

Of all the patients examined, 36 (40%) were male, 55 (60%) were female, with a female: male ratio of 1:1.4. In PG, POF and PGCG, females were more commonly affected than males, but in IF more males were affected. The distribution of fibrous and hemorrhagic lesions in relation to gender is shown in Tables 3 and 4. The relationship between gender and fibrous/hemorrhagic lesions was not statistically significant (P=0.32).

**Table 3:** Distribution of fibrous lesions by gender

**Lesion**	**IF**	**POF**	**GCF**	**EF**	**IPH**	**Total**
Gender	No. (%)	No. (%)	No. (%)	No. (%)	No. (%)	No. (%)
Male	10 (11.5)	2 (2)	1 (1)	1 (1)	1 (1)	15 (16.5)
Female	8 (8.5)	9 (10)	2 (2)	1 (1)	0	20 (21.5)
Total	18 (20)	11 (12)	3 (3)	2 (2)	1 (1)	35 (38)

IF: Irritation fibroma, POF: Peripheral ossifying fibroma, GCF: Giant cell fibroma, EF: Epulis fissuratum, IPH: Inflammatory papillary hyperplasia

**Table 4:** Distribution of soft hemorrhagic lesions by gender

**Lesions**	**PG**	**PGCG**	**PT**	**EG**	**Total**
Gender	No. (%)	No. (%)	No. (%)	No. (%)	No. (%)
Male	15 (16.5)	5 (5.5)	0	1 (1)	21 (23)
Female	22 (24.5)	10 (11)	2 (2)	1 (1)	35 (38.5)
Total	37 (41)	15 (16.5)	2 (2)	2 (2)	56 (61.5)

PG: Pyogenic granuloma, PGCG: Peripheral giant cell granuloma, PT: Pregnancy tumor, EG: Epulis granulomatosum

With 27 cases, gingiva of the lower jaw was the most common site, followed by gingiva of the upper jaw (23 cases), lip (15 cases), buccal mucosa (12 cases), and tongue (three cases). Gingiva of the upper jaw was the most common site for fibrous lesions, and these lesions were not found in tongue area. Gingiva of the lower jaw was the most common site for hemorrhagic lesions, and these lesions were not found in palate area. In IF and PG, the most common sites affected were the buccal mucosa and gingiva, respectively, but POF and PGCG were limited to the gingiva. The distribution of fibrous and hemorrhagic lesions according to location is shown in Tables 5 and 6.

**Table 5:** Distribution fibrous lesions by location

**Lesions**	**IF**	**POF**	**GCF**	**EF**	**IPH**	**Total**
location	No. (%)	No. (%)	No. (%)	No. (%)	No. (%)	No. (%)
Gingiva Max	3 (3)	6 (6.5)	0	1 (1)	0	10 (10.5)
Gingiva Man	2 (2)	3(3)	1 (1)	1 (1)	0	7 (7)
Tongue	0	0	0	0	0	0
Buccal	7 (8.5)	0	0	0	0	7 (8.5)
Palate	3 (3)	0	0	0	1 (1)	4 (4)
Lip	2 (2)	0	2 (2)	0	0	4 (4)
Total	17 (18.5)*	9 (9.5)*	3 (3)	2 (2)	1 (1)	32 (34)

IF: Irritiation fibroma, POF: Peripheral ossifying fibroma, GCF: Giant cell fibroma, EF: Epulis fissuratum, IPH: Inflammatory papillary hyperplasia

* Location of lesion was not recorded in the patient's record (one case of Irritation fibroma, two cases of peripheral ossifying fibroma)

**Table 6:** Distribution of soft hemorrhagic lesions by location

**Lesions**	**PG**	**PGCG**	**PT**	**EG**	**Total**
location	No. (%)	No. (%)	No. (%)	No. (%)	No. (%)
Gingiva Max	6 (6.5)	6 (6.5)	0	1 (1)	13 (14)
Gingiva Man	9 (10)	8 (9)	2 (2)	1 (1)	20 (22)
tongue	3 (3)	0	0	0	3 (3)
Buccal	5 (6)	0	0	0	5 (6)
Palate	0	0	0	0	0
Lip	11 (12)	0	0	0	11 (12)
Total	34 (37)*	14 (15)*	2 (2)	2 (2)	52 (56)

PG: Pyogenic granuloma, PGCG: Peripheral giant cell granuloma, PT: Pregnancy tumor, EG: Epulis granulomatosum

* Location of lesion was not recorded in the patient's record. ( 3 cases of Pyogenic granuloma, 1 case of Peripheral giant cell granuloma)

## Discussion

Reactive hyperplastic lesions are relatively common in centers of oral pathology. In the present study, the prevalence of reactive lesions was 20.2%, consistent with a report by Mashhadi Abbas et al. (7). This type of lesion also comprised 35.2%, 48%, and 39% of the total number of accessed biopsies in other studies from Iran (8-10). However, in reports by Effiom et al. (1), Reddy et al. (3), and Buchner et al. (11), reactive lesions accounted for 5.6%, 12.8%, and 6.7% of all cases. This variation in prevalence of reactive hyperplastic lesions in different countries could be due to different systems of classification and terminology of these lesions. In addition, geographical differences, as well as lifestyle and racial factors might have affected the results. 

In the present study, the most frequent lesion was PG (41%), consistent with the findings of other studies (1,8,12-14). However, in studies by Hashemi pour et al. (10), Aghbali et al. (9), Reddy et al. (3), Buchner et al. (11), and Zhang et al. (15), the most common lesion was irritation fibroma. Table 7 shows the relative frequency of oral cavity reactive lesions in different studies.

**Table 7:** Relative frequency of relative lesions of the oral cavity in various countries

**Lesion**	**FFH**	**PG**	**POF**	**PGCG**	**Total**
Study	No. (%)	No. (%)	No. (%)	No. (%)	No. (%)
Kfir et al.(16) 1980 USA	414 (55.9)	199 (26.8)	78 (10.6)	50 (6.7)	741 (100)
Stablein(17) 1985 USA	163 (35.4)	197 (42.8)	74 (16.1)	26 (5.7)	460 (100)
Macleod and Soames (18) 1987 England	48 (24)	57 (28.5)	81 (40.5)	14 (7)	200 (100)
Daley et al.(19) 1990 Canada	794 (61.2)	154 (11.9)	283 (21.8)	67 (5.1)	1298 (100)
Zang et al.(15) 2007 China	1489 (61)	482 (19.8)	431 (17.7)	37 (1.5)	2439 (100)
Zarei et al.(20) 2007 Iran	21 (18.9)	40 (36)	18 (16.2)	32 (28.9)	111 (100)
Buchner et al.(11) 2010 Israel	532 (31.8)	488 (29.1)	341 (20.4)	314 (18.7)	1675 (100)
Effiom et al.(1) 2011 Nigeria	61 (19.4)	179 (57)	64 (20.4)	10 (3.2)	314 (100)
Reddy et al.(3) 2011 India	120 (57.4)	39 (18.7)	37 (17.7)	13 (6.22)	209 (100)
Kashyap et al.(21)2013 India	35 (35)	42 (42)	18 (18)	10 (10)	100 (100)

In the present study, the frequency of hemorrhagic lesions (62%) was greater than that of fibrous lesions (38%), consistent with the results of a study by Seyedmajidi et al. (5). However, in study by Saifi et al. (8), fibrous lesions were more common (fibrous lesions, 59%; hemorrhagic lesions, (41%).

In this study, the mean age of patients with reactive lesions was 33.95±17.75 years, consistent with the reports of Al-Khateeb, Effiom et al. (1) and Reddy et al. (6,1,3). The most common age group of patients presenting with reactive lesions was 21–40 years (35%), consistent with the report of Saifiet et al. (8). In contrast, the frequency of reactive lesions was higher among individuals under 40 years of age. These lesions are not common over 70 years of age because people in this age group are largely edentulous and do not usually receive regular dental checkups. Therefore, many asymptomatic lesions are not be detected in this group (8).

The frequency of hemorrhagic lesion was higher in the 21–40 age group, while frequency of fibrous lesions was higher in the 41–60 age group. In a report by Saifi et al. (8), similar to the present study and other studies, the age of appearance of fibrous reactive lesions was higher than that of hemorrhagic lesions. This may indicate that these reactive lesions begin as hemorrhagic lesions and are gradually converted to fibrous lesions. This conversion is associated with a decrease in inflammation, an increase in the formation of fibrous tissue, and calcification of hemorrhagic lesions (8). In the present study, 60.3% of reactive lesions were found in women; the male-to-female ratio was 1:1.45. Most of the available studies (1,7,9,11,12,15) have shown a higher prevalence of lesions in women compared with men. Only Jalayer Naderi et al. (2) reported a higher prevalence of these lesions in men. This difference may be due to ethnic differences across the various studies (11). A higher prevalence of these lesions in women may show the role of hormonal factors as predisposing factors in the development of these lesions (7,8) and it could reflect a greater attention in female patients to dental care (3,7).

In many studies (2,5,7,9,10,16,20,22) the gingiva was the most common location for reactive lesions, consistent with the results of the present study. The incidence of these lesions in the gingiva indicates that reactive lesions originate from the periodontal ligament and connective tissue (2). Furthermore, it could be due to the tendency of the interdental space to aggregation of bacterial plaque and food particles which cause the gingiva expose to chronic irritation (11).

The PG is a common tumor-like growth of the oral cavity that shows a striking predilection for the gingiva. Although PG can occur at any age, it is most prevalent in children and young adults. Most studies showed a definite female predilection (5). In this study, PG (the most prevalent hemorrhagic lesion) constituted 37% of the reactive lesions among patients with an average age of 30.4±14.9 years. Females were affected more than males and the most common site was gingiva; similar findings were reported by Effiom et al. (1).The predilection of PG for women may be due to effect of female hormones. Also the gingiva is a target organ for direct function of estrogen and progesterone (1).

The irritation fibroma account for the great majority of localized reactive lesions as was substantiated by various reports in literature. Although the irritation fibroma occurs anywhere in the oral cavity; but the buccal mucosa is the most common location. These lesions are most prevalent in the 4–6^th ^decade of life, and the female-to-male ratio is almost 2:1 (5). In this study, irritation fibroma (the most common fibrous lesion) constituted 20% of the reactive lesions with among patients with an average age of 42.5±23.4 years. The most common site was the buccal mucosa, and males were affected more than females; similar to the Jalayer Naderi et al. report (2).

POF and PGCG showed a consistent pattern when compared with other studies (1,3). Unlike other oral reactive lesions they were limited to the gingiva. Eversole and Rovin indicated that being confined to the gingiva in POF and PGCG supports a histogenic derivation from the periodontal ligament (1).

In summary, in this study the prevalence of reactive lesions was 20.2%. The most common peripheral lesion was PG. The mean age of patients was 33.95±17.75 years and reactive lesions were more common in women. The most common locations of involvement were the gingiva and the alveolar mucosa of the mandible. Some of the differences observed between our study and previous studies could be attributed to racial differences and the use of different classification.

## Conclusion

Peripheral reactive lesions are a common group of lesions that may be encountered during routine dental examinations. Early detection and treatment of reactive lesions by dentists can reduce dentoalveolar complications. Therefore knowledge of the frequency and distribution of these lesions is beneficial when establishing a diagnosis and a proper treatment plan in practice.
